# Nodding Syndrome: Shifting the Focus from Cause to Treatment

**DOI:** 10.4269/ajtmh.25-0692

**Published:** 2026-03-24

**Authors:** Matthew D. Myers, Torie P. Hagin, Sanjana Ramchandran, Sally M. Yan, Riya N. Soni, Emmanuel Latim, Nilam J. Soni

**Affiliations:** ^1^ Charles E. Cheever Jr. Center for Medical Humanities & Ethics, Joe R. and Teresa Lozano Long School of Medicine, University of Texas Health San Antonio, San Antonio, Texas, USA;; ^2^ Department of Pediatrics, Gulu University, Gulu, Uganda;; ^3^ Division of Pulmonary Diseases and Critical Care Medicine, Joe R. and Teresa Lozano Long School of Medicine, University of Texas Health San Antonio, San Antonio, Texas, USA;; ^4^ Division of Hospital Medicine, Joe R. and Teresa Lozano Long School of Medicine, University of Texas Health San Antonio, San Antonio, Texas, USA;; ^5^ Medicine Service, South Texas Veterans Health Care System, San Antonio, Texas, USA

## Abstract

Nodding Syndrome (NS) is a debilitating neurodegenerative disease plaguing children in sub-Saharan Africa, with the highest prevalence in northern Uganda and South Sudan. The disease is characterized by head nodding, seizures, cognitive impairment, and physical wasting. Despite decades of research, the exact etiology of NS remains unclear. Some evidence suggests a link to *Onchocerca volvulus* infection, vitamin deficiency, and war-related displacement. Although the etiology remains unclear, effective treatment has been demonstrated with a combination of anticonvulsants, nutritional supplementation, and psychological/social therapy. Comprehensive care has been shown to slow disease progression and even reverse symptoms in some patients. NS presents complex challenges beyond its clinical scope, including stigma, caregiver burden, and political mistrust. Though researching the exact etiology of NS shall continue, sufficient resources should be allocated to implement multidisciplinary care programs and prioritize symptomatic treatment to improve the lives of children and families affected by this devastating condition.

## INTRODUCTION

Nodding Syndrome (NS) is an uncommon neurodegenerative disease affecting children in sub-Saharan Africa whose etiology has remained elusive for decades. First described in Tanzania in the 1930s, NS has been diagnosed in South Sudan (1991), Uganda (1994), Democratic Republic of Congo (2016), Cameroon (2018), Central African Republic (2019), and possibly Liberia (1983).^1^ The estimated prevalence is 0.3% in Tanzania, 0.4% in Democratic Republic of Congo, 0.7% in northern Uganda, and 4.6% in South Sudan.^2^ The disease has been most studied in northern Uganda, where an estimated 3,320 to 3,500 children have been diagnosed, with a peak incidence in 2008 after the country’s civil war.^3^^,^^4^

Although the exact etiology of NS remains a mystery, appropriate treatment regimens have been shown to halt progression and sometimes even reverse the disease. While research continues to elucidate the pathophysiology of NS, targeted programs that provide symptomatic relief to patients and reduce burden on families should be implemented. Based on a literature review and our firsthand experience in caring for NS patients, we provide a concise overview of the diagnosis and recommended treatment regimens for NS.

## DEFINITION

**Figure 1.**
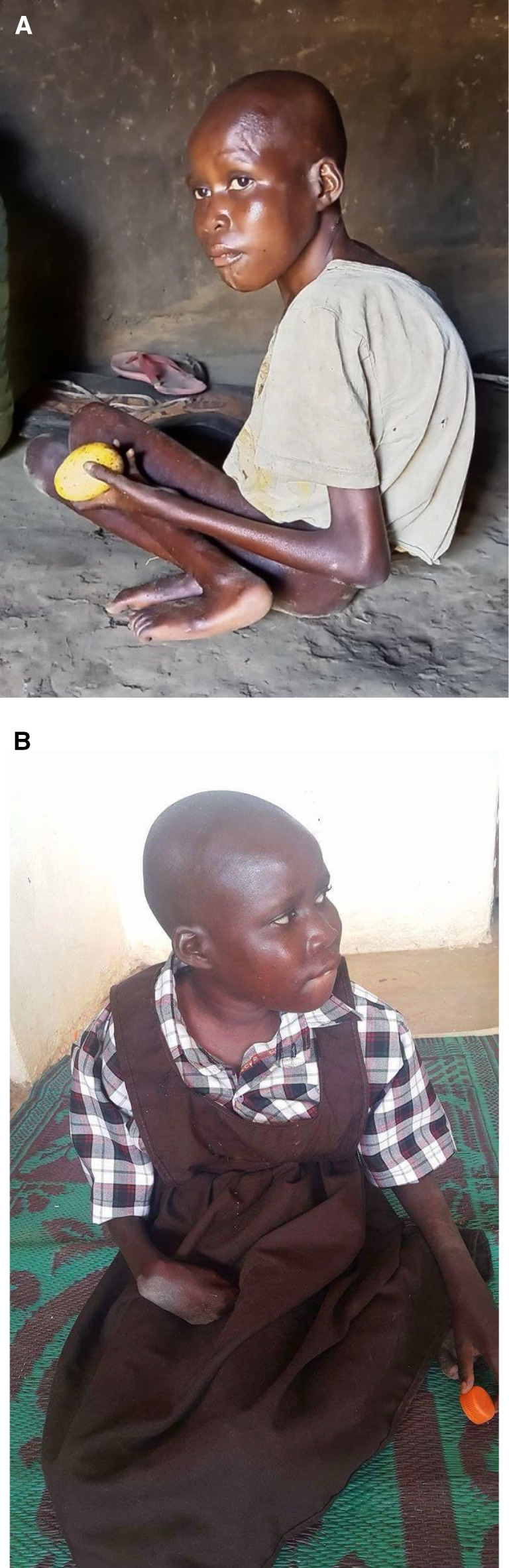
Effect of treatment on clinical features of nodding syndrome. (**A**) This previously healthy child developed stunted growth, malnutrition, seizures, and head nodding and was diagnosed with nodding syndrome. The child is shown at the time of diagnosis and (**B**) several months after treatment with nutritional supplementation, anticonvulsant medication, physical therapy, and psychiatric and social support. (Courtesy of Jolly and Emmy Okot in Gulu, Uganda, who obtained consent for publication of photos from the patient’s family).

NS is a progressive, debilitating neurodegenerative disease that affects children and is characterized by head nodding, generalized seizures, cognitive decline, stunted growth/wasting, developmental delay, and psychiatric symptoms (Figure 1).^5^ Case definitions for suspected, probable, and confirmed cases of NS were defined during the first International Scientific Meeting on NS in Uganda in 2012 (Table 1).^6^

**Table 1:** Diagnosis and management of nodding syndrome

Diagnosis
Structured case definitions with three categories: Suspected case: Previously healthy patient presenting with head nodding. Probable case: Suspected case meeting both major criteria and more than one minor criterion. Confirmed case: Probable case with head nodding that is observed and documented by a clinician, videotaped, or captured by electroencephalogram or electromyogram. Diagnostic criteria: Major criteria: 1) Age of onset between 3–18 years, 2) Frequent nodding (5–20 times/minute) Minor criteria: 1) Other neurological abnormalities (cognitive decline, behavioral problems, seizures), 2) Spatial or temporal clustering of cases, 3) Nodding triggered by food/cold temperatures, 4) Stunting or wasting, 5) Delayed sexual or physical development, and 6) Psychiatric symptoms.

Treatment
Seizure management First-line treatment: Sodium valproate (start 10 mg/kg/day divided in two doses, and increase the dose by 5–10 mg/kg/day increments until seizure control is achieved or the maximum dose is reached) Other anticonvulsants: Phenytoin (4–8 mg/kg/day), carbamazepine (10–20 mg/kg/day), phenobarbital (3–5 mg/kg/day) Status epilepticus: Intravenous or intramuscular benzodiazepines (diazepam 0.3 mg/kg, midazolam 0.3 mg/kg, or lorazepam 0.1 mg/kg)
Nutritional supplementation Vitamins B_6_ and B_12_: Daily vitamin B complex supplementation is recommended to support neurological function. Vitamin A: Single dose of vitamin A 200,000 IU is recommended for inpatient treatment of patients without edematous malnutrition. Patients with edematous malnutrition should only receive Vitamin A if a severe deficiency is apparent. Vitamin D: Vitamin D deficiency is common and is linked with anti-epileptic treatment. Supplement based on severity of deficiency. Multivitamins: General supplementation to improve body mass index and address physical stunting and wasting.
Behavioral and Psychiatric Support Behavioral interventions: Physical, language, and speech therapy; cognitive stimulation. Psychiatric support: Refer to mental health services for psychiatric medications. Consider caregiver support due to economic burden, caregiver fatigue, depression, and anxiety from caring for patients with nodding syndrome. Mood stabilization: Sodium valproate can help regulate mood; antidepressants are indicated for treatment of depression or posttraumatic stress disorder, and anxiolytics for anxiety.
Social support Special needs education: Ensure continued learning despite cognitive impairments. Therapeutic interventions: Music and dance therapy can reduce disease severity and improve social interactions. Counseling: Educate patients and caregivers on emotional, behavioral, and social problems associated with nodding syndrome; Provide follow-up care to assess solutions.
Physical Therapy Physical rehabilitation: Maintain motor function to prevent physical decline.
Other therapies Packed red blood cell transfusions: Transfuse (5–7 mL/kg) as needed for anemia. Rehydration solution: Treat undernutrition and dehydration with ReSoMal (5 mL/kg/hour for 2 hours, then 5–10 mL/kg alternating with therapeutic milk formula 75 mL every hour for up to 10 hours). Antibiotics: Treat infections with appropriate antibiotics. Wound care: Patients require frequent skin evaluation and treatment of burns and lacerations. Sexual abuse screening and treatment: Sexual abuse is more common in female patients, especially those who wander. Routine screening for abuse, as well as sexually transmitted infections, should occur with appropriate treatment and counseling of patients and their families.

## ETIOLOGY

Data from multiple studies have demonstrated an association between NS and *Onchocerca volvulus* (OV) infection. Skin snip microscopy, serology, and polymerase chain reaction (PCR) analysis have demonstrated a higher prevalence of OV infection in NS patients.^2^ OV is transmitted by the blackfly (*Simulium* spp.). Efforts to control the blackfly population include aerial spraying, ground application of larvicides along rivers, and community-based slash-and-clear methods that eliminate vegetation where blackflies breed.^7^ Studies suggest that vector control techniques, along with routine use of ivermectin to treat patients with onchocerciasis, have helped reduce the incidence of NS in Uganda.^6^^,^^8^ Some health researchers suggest that OV infection may be directly neurotoxic, whereas others hypothesize that formation of autoantibodies, such as those targeting the leiomodin-1 protein, leads to NS. Opponents of this explanation point out that cerebrospinal fluid in NS patients lacks OV genetic material and that leiomodin-1 is found mostly in smooth muscle of cerebral blood vessels and not in neurons or glia.^9^ However, RNA viruses that infect common human parasitic nematodes may elicit autoimmune reactions that lead to development of NS and other OV-related epileptic disorders.^10^ Although OV infections are found throughout South America and sub-Saharan Africa, NS has been documented only in sub-Saharan Africa, possibly due to higher microfilarial burden contributing to NS development.^11^^–^^13^ Genetic predisposition of certain human leukocyte antigen (HLA) haplotypes, particularly HLA-B35:01, may also explain the higher prevalence of NS in sub-Saharan Africa.^14^

Vitamin B_6_ deficiency has been postulated as a contributor to NS development, given its role in neurotransmitter synthesis and seizure susceptibility. Although several studies have reported low or borderline low vitamin B_6_ levels in patients with NS, similar deficiencies have been observed in unaffected family members, suggesting vitamin B_6_ is unlikely to be a primary etiological factor. Comprehensive metabolic, cerebrospinal fluid, and nutritional evaluations have failed to demonstrate a specific association between vitamin B_6_ deficiency and NS. Moreover, vitamin B_6_ metabolites found in the cerebrospinal fluid of NS patients have been in normal ranges. Taken together, current evidence supports vitamin B_6_ deficiency as a common comorbidity rather than a causative factor of NS. Nonetheless, given the high prevalence of nutritional deficiencies in affected regions and the established role of vitamin B_6_ in neurological function, vitamin B_6_ supplementation remains a reasonable supportive intervention for NS patients.^15^

Regarding the etiology of NS, associations have also been drawn between NS and internal displacement camps during the Ugandan civil war, mycotoxins from Amanita mushroom species, such as *Amanita bingensis*, and malnutrition.^2^ Postmortem histological analysis of NS patients revealed tau protein deposits in the cerebral cortex, brainstem, and basal ganglia. Gliosis and psammoma bodies have also been observed surrounding the choroid plexus.^13^^,^^16^ However, causal evidence linking these findings to NS is lacking to date.

## CURRENT AND FUTURE TREATMENTS

As research continues to better understand the pathophysiology of NS, patients and their families should be offered symptomatic and supportive treatments. Ideally, a multidisciplinary team of specialists would develop a comprehensive treatment plan that integrates pharmacological interventions, nutritional support, behavioral therapies, and physical rehabilitation. Table 1 lists the most common current treatment recommendations.^2^^,^^6^

## INVESTIGATIONAL AND PREVENTIVE THERAPIES

Significant research has been conducted to find new treatments for NS. A study conducted in northern Uganda assessed the efficacy of a 6-week course of doxycycline and demonstrated a significant decrease in seizure-related hospitalizations and deaths among NS patients who received doxycycline. The study concluded that doxycycline can be considered an adjunct to antiseizure medication to reduce the risk of fatal complications from acute seizures.^17^ Doxycycline is known to kill a parasite called *Wolbachia*, which is an obligate OV endosymbiont and therefore present in most infections.^18^ In addition, control of OV infection through community-guided treatment with ivermectin, larviciding of rivers, and aerial spraying has been associated with reduced incidence of NS, even though a direct correlation cannot be proven.^2^ Since the 2012 International Scientific Meeting on NS, the WHO has officially recommended ivermectin as a preventive therapy for NS.^6^^,^^19^

## BENEFITS OF COMPREHENSIVE CARE

A comprehensive treatment plan, including nutritional supplementation, medications, and physical and cognitive therapies, has been shown to produce significant improvement of NS patients. An example of a successful center providing comprehensive care was Hope for Humans, a clinic in northern Uganda that provided care to >40 NS patients between 2012 and 2017.^20^ The improvement in NS patients was striking. Not only was disease progression halted, but several patients showed improvement beyond baseline. The Hope for Humans clinic set a fantastic precedent for comprehensive care of NS patients. Unfortunately, the clinic was forced to close when private funding and donations were exhausted, and several patients experienced regression to prior clinical states.

Although comprehensive, multidisciplinary care is unquestionably the best approach, most NS patients reside in resource-limited settings where this approach is unsustainable. Currently, most NS patients receive care from nonphysician clinicians, primarily community health workers (CHWs), who play a crucial role in providing longitudinal care. Perhaps the most important role of CHWs is ensuring anticonvulsant medications reach NS families, even in remote regions. However, the current approach to prescribing medications tends to follow a one-size-fits-all model, with prolonged use of low-dose anticonvulsant medications and minimal clinical follow-up, resulting in ineffective seizure control for many patients. Beyond pharmacotherapy, minimal government aid is provided to NS patients and their families. The inadequacy of the current model of NS care underscores the importance of developing a more comprehensive framework through which patient’s physical, mental, emotional, and social needs can be addressed. Telemedicine is one potential solution to connect NS patients in remote settings to specialty care at referral centers.

## SOCIAL, POLITICAL, AND ECONOMIC CHALLENGES

Beyond its clinical features, addressing NS entails complex social, political, and economic challenges. The emergence of NS in northern Uganda during the 1990s is deeply linked to the country’s civil war between the Ugandan government and the Lord’s Resistance Army (1991–2006).^1^^,^^21^ Long-standing distrust of the government, caused by ethnic division and war, shaped local responses to NS. Uncertainty about the disease’s etiology fueled suspicions and rumors, including fears of the government specifically targeting the Acholi community, the primary ethnic group affected by NS. Distrust of both the government and international researchers further undermined community acceptance of interventions, which hindered implementation of effective treatments, demonstrating the lasting impact that politicization can have on public health.^21^

The costs of treating NS are significant. The Ugandan government contributes approximately 133 million Ugandan shillings (approximately USD $36,000) annually toward NS treatment.^8^ Individual families bear an immense financial burden for NS care, with an average of 8% of a family’s annual spending allotted to NS care.^22^ Cost calculations most likely underestimate the actual costs.

Limited community awareness of the signs and symptoms of NS, such as seizures and physical/intellectual disability, often results in social exclusion, exacerbating the psychological burden on patients and their families.^21^ Some communities believe that NS can spread via person-to-person transmission, which is used to justify separation of eating/sleeping quarters of NS patients from the rest of the family or community. In addition, some individuals with NS have experienced sexual abuse, exploitation, and forced child labor. Caregivers frequently endure sleep deprivation, depression, and stress, which can result in substance abuse, domestic violence, and even suicidal and homicidal thoughts.^2^

Public health campaigns can disseminate information on approaches to reduce the incidence of NS, such as ivermectin treatment and black fly eradication, and help dispel misinformation, such as person-to-person transmission. Furthermore, demystifying the pathogenesis of NS can potentially reduce stigmatization of patients and their families and rebuild trust in public health efforts. Dismantling years of accumulated negative stigma and distrust paves the way for more effective support and care of NS patients in the future.

## CONCLUSION

NS is a challenging disease from both scientific and humanitarian perspectives. The ambiguous etiology of the disease paired with Uganda’s political, social, and economic conditions has hindered research and treatment efforts. Although continued investigation into the pathophysiology of NS remains essential, the primary clinical imperative should focus on delivery of comprehensive, sustained care of patients with NS. Multidisciplinary management, including nutritional supplementation, appropriate antiseizure medications with optimized dosing, and physical and cognitive therapies, should be prioritized to improve patient outcomes when feasible. However, the effectiveness of even the most carefully designed treatment plans is ultimately constrained by access to consistent care and medications. Intermittent or limited availability of therapy substantially diminishes potential benefits. Therefore, efforts to improve outcomes must focus not only on individualized care plans but also on ensuring long-term, reliable access to care.

## References

[1] Spencer PS Okot C Palmer VS Valdes Angues R Mazumder R, 2022. Nodding syndrome: A key role for sources of nutrition? eNeurologicalSci 27: 100401. 35480298 10.1016/j.ensci.2022.100401 PMC9035392

[2] Abd-Elfarag GOE Edridge AWD Spijker R Sebit MB van Hensbroek MB, 2021. Nodding syndrome: A scoping review. Trop Med Infect Dis 6: 211. 34941667 10.3390/tropicalmed6040211 PMC8703395

[3] Idro R Opar B Wamala J Abbo C Onzivua S Mwaka DA Kakooza-Mwesige A Mbonye A Aceng JR, 2016. Is nodding syndrome an *Onchocerca volvulus*-induced neuroinflammatory disorder? Uganda’s story of research in understanding the disease. Int J Infect Dis 45: 112–117. 26987477 10.1016/j.ijid.2016.03.002

[4] Burton A, 2016. Uganda: How goes the nodding syndrome war? Lancet Neurol 15: 30–31. 26700904 10.1016/S1474-4422(15)00350-6

[5] Abd-Elfarag GOE Mathewson JD Emmanuel L Edridge AWD van Beers S Sebit MB Colebunders R van Hensbroek MB Rood EJJ, 2023. Nodding Syndrome: Clinical characteristics, risks factors, access to treatment, and perceptions in the Greater Mundri Area, South Sudan. Pathogens 12: 190. 36839462 10.3390/pathogens12020190 PMC9965143

[6] Idro R, , 2013. Proposed guidelines for the management of nodding syndrome. Afr Health Sci 13: 219–232. 24235917 10.4314/ahs.v13i2.4 PMC3824512

[7] Jada SR, , 2023. Effect of onchocerciasis elimination measures on the incidence of epilepsy in Maridi, South Sudan: A 3-year longitudinal, prospective, population-based study. Lancet Glob Health 11: e1260–e1268. 37474232 10.1016/S2214-109X(23)00248-6

[8] Aceng JR, 2018. Statement on Nodding Syndrome in Northern Uganda. Press release. March 5, 2018. https://www.health.go.ug/download/file/fid/1800. Accessed January 13, 2025.

[9] Kodja KG Onzivua S Kitara DL Fong A Kim P Pollanen MS, 2023. Nodding syndrome is unlikely to be an autoimmune reaction to leiomodin-1 after infection by Onchocerca volvulus. Biochem Biophys Rep 35: 101498. 37601452 10.1016/j.bbrep.2023.101498 PMC10439352

[10] Quek S, , 2024. Diverse RNA viruses of parasitic nematodes can elicit antibody responses in vertebrate hosts. Nat Microbiol 9: 2488–2505. 39232205 10.1038/s41564-024-01796-6 PMC11445058

[11] Chesnais CB, , 2020. A second population-based cohort study in Cameroon confirms the temporal relationship between onchocerciasis and epilepsy. Open Forum Infect Dis 7: ofaa206. 32587878 10.1093/ofid/ofaa206 PMC7304933

[12] Colebunders R Njamnshi AK Menon S Newton CR Hotterbeekx A Preux PM Hopkins A Vaillant M Siewe Fodjo JN, 2021. Onchocerca volvulus and epilepsy: A comprehensive review using the Bradford Hill criteria for causation. PLoS Negl Trop Dis 15: e0008965. 33411705 10.1371/journal.pntd.0008965 PMC7790236

[13] Hadermann A Amaral LJ Van Cutsem G Siewe Fodjo JN Colebunders R, 2023. Onchocerciasis-associated epilepsy: An update and future perspectives. Trends Parasitol 39: 126–138. 36528471 10.1016/j.pt.2022.11.010

[14] Hadermann A, , 2025. Investigating HLA haplotypes as a potential risk factor for nodding syndrome: A case-control study in the Mahenge area, Tanzania. PLoS Negl Trop Dis 19: e0012971. 41296809 10.1371/journal.pntd.0012971 PMC12680330

[15] Soldatos A, , 2023. Genomic analysis, immunomodulation and deep phenotyping of patients with nodding syndrome. Brain 146: 968–976. 36181424 10.1093/brain/awac357 PMC10169415

[16] Hotterbeekx A Lammens M Onzivua S Lukande R Olwa F Kumar-Singh S Van Hees S Idro R Colebunders R, 2021. Neuropathological changes in Nakalanga Syndrome – A case report. Pathogens 10: 116. 33498763 10.3390/pathogens10020116 PMC7912209

[17] Idro R, , 2024. Doxycycline for the treatment of nodding syndrome: A randomised, placebo-controlled, phase 2 trial. Lancet Glob Health 12: e1149–e1158. 38754459 10.1016/S2214-109X(24)00102-5 PMC11191365

[18] Walker M Specht S Churcher TS Hoerauf A Taylor MJ Basáñez MG, 2015. Therapeutic efficacy and macrofilaricidal activity of doxycycline for the treatment of river blindness. *Clin Infect Dis* 60: 1199–1207. 25537873 10.1093/cid/ciu1152 PMC4370165

[19] World Health Organization Eastern Mediterranean Region, 2012. *Nodding Syndrome: A Devastating Illness*. Available at: https://www.emro.who.int/media/news/nodding-syndrome.html. Accessed December 23, 2025.

[20] Gazda S Kitara DL, 2018. Treatment and rehabilitation outcomes of children affected with nodding syndrome in northern Uganda: A descriptive case series. Pan Afr Med J 29: 228. 30100981 10.11604/pamj.2018.29.228.13627 PMC6080981

[21] Irani J Rujumba J Mwaka AD Arach J Lanyuru D Idro R Gerrets R Grietens KP O’Neill S, 2019. “Those who died are the ones that are cured." Walking the political tightrope of Nodding Syndrome in northern Uganda: Emerging challenges for research and policy. PLoS Negl Trop Dis 13: e0007344. 31220081 10.1371/journal.pntd.0007344 PMC6605670

[22] Latio LSY, , 2020. Economic burden of the persistent morbidity of nodding syndrome on caregivers in affected households in Northern Uganda. PLoS One 15: e0238643. 32991607 10.1371/journal.pone.0238643 PMC7523991

